# Early Comprehensive Geriatric Assessment in Acute Care: Evidence and Implementation

**DOI:** 10.7759/cureus.112760

**Published:** 2026-07-16

**Authors:** Eve Ducker

**Affiliations:** 1 Acute Medicine, Royal Cornwall Hospitals Trust, Truro, GBR

**Keywords:** acute care medicine, acute medical admissions, clinical frailty, comprehensive geriatric assessment, holistic care for patients, morbidity and frailty index, multidisciplinary care team

## Abstract

The growing prevalence of frailty among older adults presenting to acute care settings poses significant challenges for healthcare systems worldwide. Frail older patients are at increased risk of adverse outcomes, including delirium, functional decline, prolonged hospitalisation and mortality. Comprehensive Geriatric Assessment (CGA), a multidimensional and interdisciplinary approach to evaluating medical, functional, psychological, and social needs, has emerged to tackle some of these challenges.

Despite its central role in evaluating frail patients, CGA is often only introduced after admission. Earlier recognition of frailty may support more individualised decision-making, better alignment of care with patient needs, and closer collaboration across multidisciplinary teams. This editorial argues that CGA should be initiated as early as possible within the acute care pathway, ideally beginning in emergency departments and acute medical units. This is considered alongside the challenges of implementing CGA, including workforce pressures, service configuration, and access to specialist expertise.

As healthcare systems adapt to population ageing, earlier incorporation of CGA offers an opportunity to reshape the delivery of acute care for older adults.

## Editorial

The ageing UK population has led to a growing prevalence of frailty among older adults, resulting in an increasing number of patients presenting to acute care settings with complex multimorbidity, functional impairment, and geriatric syndromes [1,2]. These patients frequently experience adverse outcomes during hospitalisation, including delirium, functional decline, prolonged length of stay, institutionalisation, and increased mortality [3,4]. Traditional acute care models, largely organised around single-disease frameworks, are often insufficient to address the holistic needs of this population.

Comprehensive Geriatric Assessment (CGA) is a structured, multidisciplinary approach that evaluates medical, functional, pharmaceutical, psychological, and social domains to guide individualised care planning. It involves input from not only physicians but other members of the multidisciplinary team such as occupational therapists, physiotherapists, pharmacists and social care workers. Evidence from systematic reviews has demonstrated that CGA improves outcomes for frail or elderly inpatients [1]. However, in many healthcare systems, CGA is introduced late during admission or inconsistently applied, limiting its potential benefit [3,4].

There is growing research and recognition that earlier identification of frailty and timely implementation of CGA within acute care pathways improves outcomes and reduces avoidable harm [1,3,4]. The evidence supporting early CGA is robust and expansive; this editorial will outline some of the key pieces of research, including systematic reviews, meta-analyses and novel research.

Randomised controlled trials demonstrated improved outcomes for older inpatients receiving CGA compared with standard care. Landefeld et al. showed that structured geriatric evaluation improved survival and functional outcomes in activities of daily living [5].

More recent research has strengthened this evidence. The systematic review by Ellis et al. (2017) included 13,766 participants from 29 randomised controlled trials and found that patients receiving CGA were more likely to be alive and living at home at follow-up, with reduced institutionalisation [1]. Across studies, CGA is associated not only with improved survival and independence but also with care that is more aligned with patient-centred goals. Collectively, this evidence establishes CGA as the gold-standard model of care for frail older adults in acute hospital settings. The implementation of CGA at the front door has been proven to be essential; with the use of frailty scoring systems such as the Rockwood frailty score, this could help identify which patients will benefit the most from CGA.

A key element of the CGA is early implementation; this is because frail patients deteriorate quickly in the hospital environment, with lasting negative impacts on their health. Keeble et al. demonstrated that older adults with frailty discharged following brief admissions of less than 72 hours experienced significantly increased 2-year mortality and healthcare utilisation compared with non-frail counterparts, highlighting the speed and severity of deterioration in this population [2]. To show how essential early CGA is, Graf et al.’s systematic review showed that CGAs performed in emergency departments improved patients’ outcomes [3].

What Graf et al. also highlighted in their review was the challenges of the timely nature of CGA in acute settings such as the emergency department [3]. This raises concerns that CGA may be delayed or incompletely implemented when embedded within already pressured emergency systems.

However, this is not the only challenge of CGA implementation. A consistent barrier described across the literature is workforce and resource limitation. CGA is a complex, multidisciplinary intervention requiring coordinated input from geriatric physicians, nursing, physiotherapy, occupational therapy, pharmacy, and social care. Systematic reviews have identified shortages in trained geriatric staff and lack of integrated multidisciplinary teams as key constraints to effective implementation in acute hospital settings [4].

Comprehensive Geriatric Assessment (CGA) remains one of the most evidence-based interventions available for improving outcomes among frail older adults in acute care settings. Robust evidence demonstrates its ability to preserve functional independence, reduce institutionalisation, and support patient-centred care. However, as populations continue to age and the prevalence of frailty rises, the challenge is no longer establishing whether CGA works but ensuring that it is delivered effectively and consistently.

Increasing evidence suggests that the greatest opportunity for impact lies in the early stages of the acute care pathway. Early identification of frailty and timely implementation of CGA may help prevent avoidable deterioration, facilitate proactive multidisciplinary management, and improve care planning from the point of admission. Despite recognised barriers, including workforce constraints, organisational fragmentation, and pressures within emergency and acute medical services, these challenges should not deter implementation efforts.

Future healthcare models must move beyond viewing CGA as a specialist intervention delivered later in admission and instead embed its principles at the front door of acute care. Achieving this shift will require investment, training, and system-level redesign, but the potential benefits for patients, healthcare systems, and society are substantial.
